# Facteurs de Gravité de L'aspergillome Pulmonaire en Réanimation Chirurgicale à L'hôpital Joseph Ravoahangy Andrianavalona, Antananarivo, Madagascar

**DOI:** 10.48327/E9QE-PV02

**Published:** 2021-02-18

**Authors:** N.N.M. Razafimanjato, J.M. Rakotoson, M. Ravoatrarilandy, R.L. Andrianasolo, A.T. Rajaonera, N.E. Raveloson, H.J.L. Rakotovao

**Affiliations:** 1 USFR de chirurgie thoracique, Hôpital universitaire Joseph Ravoahangy Andrianavalona (HUJRA), Faculté de médecine d'Antananarivo, Madagascar; 2 USFR d'anesthésie-réanimation chirurgicale, Hôpital universitaire Joseph Ravoahangy Andrianavalona (HJRA). Faculté de médecine d'Antananarivo, Madagascar; 3 USFR de maladie infectieuse et tropicale, Hôpital universitaire Joseph Raseta Befelatanana (HJRB). Faculté de médecine d'Antananarivo, Madagascar; 4 USFR de réanimation médicale, Hôpital universitaire Joseph Raseta De Befelatanana (HJRB). Faculté de médecine d'Antananarivo, Madagascar

**Keywords:** Aspergillome, Chirurgie thoracique, Lobectomie, Pneumonectomie, Soins intensifs, Réanimation, Facteurs de sévérité, Tabagisme, Tuberculose, Hôpital, Antananarivo, Madagascar, Océan Indien, Aspergilloma, Thoracic surgery, Lobectomy, Pneumonectomy, Intensive care, Resuscitation, Severity factors, Smoking, Tuberculosis, Hospital, Antananarivo, Madagascar, Indian Ocean

## Abstract

**Objectifs:**

Cette étude rétrospective a pour objectif de recueillir les données épidémio-cliniques, de décrire le profil évolutif et enfin de recenser les facteurs de gravité de la prise en charge post opératoire, en milieu de réanimation chirurgicale, de la chirurgie de l'aspergillome pulmonaire.

**Patients et Méthodologie:**

Nous avons étudié les résultats post opératoires de 34 patients ayant été opérés entre juin 2009 et juin 2014, puis admis dans le service de réanimation chirurgicale du Centre hospitalier universitaire Joseph Ravoahangy Andrianavalona (CHU-JRA).

**Résultats:**

Nous avons recensé 23 hommes et 11 femmes avec un âge moyen de 42 ± 9,9 ans. Dans 29% des cas, il s'agissait d'un aspergillome complexe. Le geste chirurgical consistait en une résection segmentaire ou atypique (n = 3), une lobectomie (n = 21), une bi-lobectomie (n = 2), une pneumonectomie (n = 7) et une spéléotomie (n = 1).

La durée de séjour en réanimation était de 4,5 ± 3 jours. Le taux de létalité global était de 15%, liée à une détresse respiratoire, au sepsis et aux pathologies sous-jacentes. Les principales complications post-opératoires étaient la pneumopathie, l'oedème aigu du poumon et l'hémorragie.

**Conclusion:**

Les facteurs associés à une complication majeure étaient le score ASA, une altération préopératoire de la fonction respiratoire, la chirurgie en urgence, la poursuite du tabagisme, la ventilation mécanique post-opératoire, le saignement, une hyperleucocytose et la durée de l'intervention chirurgicale.

## Abstract (en)

**Objective:**

This retrospective study was designed to study the clinical profile, post-operative outcome with analysis of severity factors of pulmonary aspergilloma operated in our institute.

**Patients and methodology:**

During a 5-year period (June 2009 to June 2014), 34 patients underwent surgery for pulmonary aspergilloma and were admitted in the surgical intensive care unit of the CHU/JRA Antananarivo.

**Results:**

The group consist of 23 male patients and 11 female patients with a mean age of 42 ± 9.9 years. In 29.4% of cases, it was a complex aspergilloma. The procedures performed were segmental resection (n = 3), lobectomy (n = 21), bi-lobectomy (n = 2), pneumonectomy (n = 7) and cavernostomy (n = 1). Median hospital stay was 4.5 ± 3 days. The postoperative lethality rate was 14.7% caused by respiratory failure, infection and underlying diseases. Main complications included bleeding, respiratory failures, and pulmonary infection.

**Conclusion:**

Factors associated to increased postoperative complications included: the ASA score, decreased preoperative lung function, urgent procedures, smoking, postoperative mechanical ventilation, bleeding, hyperleukocytosis and surgery duration.

## Introduction

L'aspergillome est la colonisation d'une cavité pulmonaire, bronchique, voire pleurale préexistante par une moisissure ubiquitaire appelée Aspergillus. Elle est le fait d'une diminution de l'immunité locale au niveau de la cavité, d'une modification de sa muqueuse et de la présence d'une bronche de drainage [17].

La chirurgie de l'aspergillome pulmonaire est considérée comme un défi technique pour les chirurgiens et anesthésistes-réanimateurs en raison du haut risque de complications péri-opératoires, encore plus élevé dans un centre à faible ressource. La chirurgie reste cependant le traitement de référence de cette pathologie. La prise en charge péri-opératoire doit commencer par la recherche des facteurs de risque, afin de bien sélectionner les patients qui vont bénéficier d'une exérèse pulmonaire. Les enjeux sont surtout les décompensations cardio-vasculaires et respiratoires dans le cadre de l'urgence [1, 2].

La chirurgie de l'aspergillome se pratique depuis de nombreuses années à Madagascar sans qu'aucune étude n'ait analysé les facteurs de gravité pré et peropératoires de cette pathologie. L'identification de ces facteurs de gravité permettrait d'anticiper et d'appréhender la survenue des complications postopératoires.

L'objectif de cette étude était de décrire les caractéristiques épidémiologiques et cliniques des aspergillomes pulmonaires et d'identifier les facteurs de risque pour la chirurgie en période postopératoire immédiate dans le service de réanimation chirurgicale du Centre hospitalier universitaire Joseph Ravoahangy Andrianavalona (CHU-JRA).

## Patients et Méthodes

Une étude rétrospective a été menée parmi les patients opérés d'un aspergillome pulmonaire, puis admis dans le service de réanimation chirurgicale du CHU-JRA sur une période de cinq années, entre juin 2009 et juin 2014. Les données cliniques ont été obtenues à partir des dossiers médicaux et des registres de ce service.

Ont été inclus dans l'étude, les patients ayant bénéficié d'une chirurgie de résection pulmonaire pour une greffe aspergillaire quel que soit leur âge. Le diagnostic d'aspergillome pulmonaire avait été retenu sur des faisceaux d'arguments anamnestiques, cliniques et paracliniques. La confirmation diagnostique avait été donnée par l'examen anatomopathologique de la pièce de résection pulmonaire. Les patients n'ayant pas bénéficié d'une résection pulmonaire ont été exclus.

Les paramètres préopératoires étudiés étaient l'âge des patients, l'évaluation de leur état général selon la classification de l'American Society of Anesthesiologists (ASA), leurs comorbidités et leur capacité fonctionnelle respiratoire. Une bonne tolérance à l'effort était définie par une dyspnée survenant lors d'efforts importants ou après l'ascension de plus d'un étage (stade 1 de la classification de Sadoul). Une mauvaise tolérance à l'effort correspondait aux dyspnées de stade 2 ou plus selon la classification de Sadoul [14].

En post opératoire, plusieurs paramètres ont pu être évalués, comme les complications post-opératoires majeures de grade 2 ou plus selon la classification de Clavien (pneumopathies, insuffisance respiratoire aiguë, atélectasies, fistules broncho-pleurales, embolies pulmonaires, bronchospasmes et arythmies) [12].

Les résections pulmonaires ont été analysées. Leur étendue était fonction de la nature simple ou complexe de la lésion aspergillaire. Une lésion aspergillaire était qualifiée de simple si elle était unique, localisée seulement dans un lobe pulmonaire, arrondie ou ovalaire, bien limitée, dense et homogène, ne prenant pas le contraste, occupant la partie déclive d'une cavité pulmonaire. Elle était dite complexe, si elle était multifocale ou bilatérale, avec des parois épaissies émettant des spicules dans le parenchyme adjacent, ou avec un niveau hydro-aérique.

Les indications de transfusion sanguine étaient portées en fonction de l'évaluation préopératoire et du calcul de la perte sanguine en per-opératoire. Lorsqu'elle était indiquée, la ventilation mécanique était poursuivie jusqu'à l'éveil post-opératoire.

Les paramètres postopératoires étudiés incluaient la durée du séjour en réanimation chirurgicale, l'incidence des complications post-opératoires majeures, ainsi que l'issue des patients. En raison des difficultés du suivi des patients à l'issue de leur hospitalisation, seuls les facteurs de risque de morbi-mortalité en post-opératoire immédiat ont été étudiés.

L'analyse statistique a été effectuée avec le logiciel R*3.0.0. Le test chi-carré avec correction de Yates et le test exact de Fisher, pour un effectif réduit, ont été utilisés pour l'étude comparative des valeurs des variables. Une différence a été considérée comme significative pour un p<0,05.

## Résultats

Durant la période étudiée 34 patients ont été inclus après avoir été opérés d'un aspergillome pulmonaire, soit une prévalence hospitalière de 7 cas par an. Il y avait 23 hommes et 11 femmes (sex-ratio: 2,1) ayant un âge moyen de 42 ±9,9 ans.

Les caractéristiques démographiques ainsi que l'évaluation préopératoire des patients sont représentées dans le tableau 1. La majorité des patients était classée ASA 1 ou 2 (91%). La bronchopneumopathie chronique obstructive (BPCO) et la cardiopathie ischémique sont les facteurs de co-morbidités les plus retrouvés. Seuls 23% avait une mauvaise tolérance à l'effort lors du bilan respiratoire pré opératoire. Le VEMS ppo était à plus de 40% chez la majorité des patients (85%).

**Tableau I:** Caractéristiques démographiques et évaluation préopératoire des patients Patient demographic characteristics and preoperative assessment

	Total Moy±DS	Survivants	Mortalités	Valeur p
**Age**	34 (42 ± 8 ans)	29 (85%)	5 (15%)	
**Hommes/Femmes**	23/11	20 (87%)/9	3(13%)/2	1,000
**Comorbidités**				
cardiopathies ischémiques	3	2		1,000
BPCO	5	2	3	0,573
**Classification ASA**				
ASA 1 ou 2	31	28	3	
ASA 3 et plus	3	1	2	0,050
**Fonction respiratoire préopératoire**				
bonne tolérance à l'effort	26	25	1	
mauvaise tolérance à l'effort	8	4	4	0,007
VEMS ppo > 40%	29	29	0	0,005

**Fig. 1:**
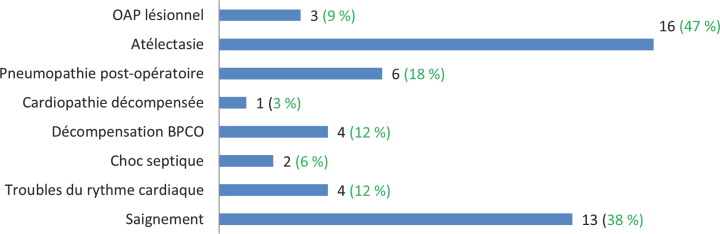
Incidence des complications post-opératoires Incidence of postoperative complications

Les différents types d'intervention sont résumés dans le tableau 2. Les formes complexes ont bénéficié de pneumonectomies, résections segmentaires et spéléotomie dans respectivement 20%, 6% et 3% des cas. La chirurgie réalisée en urgence pour problème d'hémoptysie a eu un taux de mortalité de 100% dans les premières 48 heures post opératoires. La durée moyenne de séjour en unité de soins intensifs était de 4± 2,6 jours. Les complications post-opératoires sont représentées sur la figure 1. Celles-ci étaient dominées par les atélectasies, l'hémorragie et les pneumopathies. Cinq décès sont survenus, dont 2 durant les 48 premières heures suivant la chirurgie, soit un taux de létalité globale de 15%. Il s'agissait de trois défaillances respiratoires liées à une VEMS ppo<40% ou à un oedème pulmonaire lésionnel, d'un choc septique à point de départ pulmonaire (pneumopathie postopératoire) dans un cas et d'une décompensation de cardiopathie ischémique.

**Tableau II:** Mortalité en fonction du geste chirurgical réalisé Mortality according to the surgical procedure performed

	Total n (%)	Mortalité <48 H	Mortalité >48 H	Valeur p
**Résection segmentaire**	3 (9%)	0	0	1,000
**Lobectomie**	21 (62%)	1	1	0,348
**Bilobectomie**	2 (6%)	0	0	1,000
**Pneumonectomie**	7 (21%)	1	1	0,559
**Spéléotomie**	1 (2%)	0	1	0,147
**Chirurgie**				
urgente	2 (6%)	2		0,018*
réglée	32 (94%)	1	2	

* p<0,05

Les facteurs de gravité identifiés (indépendants des complications majeures) étaient un tabagisme actif, une hyperleucocytose, la durée de l'intervention, le saignement per opératoire, ainsi que la nécessité d'une ventilation mécanique en post-opératoire (Tableau 3).

**Tableau III:** Facteurs associés à une complication postopératoire majeure Associated factors with a major post-operative complication

	Total n (%)	Complications majeures	Valeur p
**Tabagisme actif**	23 (68%)	12	0,024*
**Numération GB > 10000**	11 (32%)	7	0,026*
**Durée de l'intervention >3h**	6 (18%)	5	0,014*
**Saignement per opératoire > 1000 ml**	13 (38%)	8	0,025*
**Ventilation mécanique postopératoire**	9 (26%)	7	0,004*

* p<0,05

## Discussion

L'âge moyen et le sexe ratio des patients de cette série sont comparables à ceux d'une série algérienne rapportée par Zait et al [17] qui rapportait 39 cas de patients porteurs d'un aspergillome pulmonaire avec un âge moyen de 49,5 ans et un sex-ratio de 1,2. Comme dans les études algériennes [17] et sénégalaises [1], la quasi-totalité de nos patients présentait un antécédent de tuberculose pleuro-pulmonaire pour laquelle ils avaient été traités et déclarés guéris avant la chirurgie. Ces taux sont supérieurs à ceux retrouvé par Babatasi et al [2] en France qui notait que 60% des patients étaient d'anciens tuberculeux. Cela tient à une incidence de la maladie tuberculeuse plus faible dans les pays occidentaux, malgré l'augmentation de l'incidence co-infection VIH et tuberculose pleuro-pulmonaire. La corrélation entre le développement de la truffe aspergillaire et les lésions caverneuses séquellaire d'une infection tuberculeuse est démontrée par plusieurs auteurs [2, 14, 17]. Le délai moyen d'apparition de l'aspergillome est de 8ans après une tuberculose pulmonaire selon Rakotoson et al [14].

Dans leur série de 33 patients avec aspergillome complexe, Lejay et al [12] en France, ont établi que dans les premières 48 heures post-opératoire, il y a eu peu de mortalité, moins de saignement, et moins de complications pleurales (5%, 44% et 47% respectivement). Au-delà de 48 heures, la mortalité est nulle, le saignement à 1,9% et les complications pleurales à 18% [12]. Ceci s'explique plutôt par l'avancée des techniques de réanimation et de la modalité anesthésique que par le type de chirurgie.

Le choix du type de chirurgie est fonction du caractère simple ou complexe de la lésion; la distinction radio-clinique entre aspergillome simple et complexe n'a pas eu de valeur pronostique dans notre série. Ceci pourrait s'expliquer par le nombre moins élevé des lésions complexes opérées (29%) dans notre population d'étude. Farid et al [7] au Maroc ont opéré 30 patients présentant 40% de formes simples et 60% d'aspergillomes complexes sans aucun décès dans les 30 jours post-opératoires. Néanmoins, ils ont déploré 4 décès au cours du suivi ultérieur. Leur morbidité post-opératoire était représentée par une fuite aérienne prolongée, un empyème, une reprise chirurgicale pour une hémorragie, une détresse respiratoire nécessitant une trachéotomie et une ré intubation et une fistule broncho-pleurale. Sur 35 cas d'aspergillome complexe, dont 37,1% ont bénéficié d'une pneumonectomie et 54,2% d'une lobectomie, Ade et al [1] décrivent des suites opératoires compliquées dans 51,4% des cas, avec des hémorragies post-opératoires, une suppuration pariétale, un empyème, et le classique oedème aigu du poumon, sans décès post-opératoire immédiat. Comparativement aux données de la littérature, cette série comporte une forte proportion de pneumonectomie (20,5%) expliquant un taux de saignement élevé [1, 7]. Chatzmichalis et al [6] ont également observé un saignement excessif chez 60% des patients opérés pour aspergillome complexe. Brik et al [4] rapportent 28,2% de complications post-opératoires avec des fuites d'air prolongées, saignement, infection pulmonaire, empyème, fistule broncho-pleurale et chylothorax chez 42 patients opérés en Egypte. Les complications respiratoires (atélectasie et pneumopathie) notées dans cette série ont liées très probablement à une fraction inspirée en oxygène élevée en per et post-opératoire par le manque de moyens de mesure de ce paramètre et par la disponibilité limitée de la gazométrie artérielle en urgence dans notre centre.

La dénutrition, l'altération préalable de la fonction respiratoire par la tuberculose et le tabagisme sont d'autres facteurs expliquant ces complications [10, 16]. Le taux de mortalité dans cette série (15%) est dans la moyenne de la littérature, et tient surtout à la décompensation de pathologies préexistantes, notamment cardio-vasculaires.

La chirurgie de l'aspergillome pulmonaire est en effet grevée d'une mortalité hospitalière très variable, secondaire à la décompensation des complications liées au saignement, au sepsis et à une altération de la fonction respiratoire en post-opératoire [5, 15]. Les facteurs de gravité identifiés dans ce travail avaient également été identifiés dans des études récentes. Ces dernières retrouvaient également la préexistence d'un empyème, la latéralité droite de la lésion, une reprise chirurgicale pour hémorragie, ainsi que l'âge avancé du patient qui compromettent de façon non négligeable la survie des opérés. Malgré le faible effectif de cette cohorte, nos résultats rejoignent ceux de Stephan et al [16] qui retrouvaient trois éléments prédictifs de survenue de complications post-opératoires sur une série de 266 patients: un score ASA ≥ 3, un temps opératoire ≥ 80 min et la nécessité de ventiler mécaniquement le patient plus de 48 heures en post-opératoire.

Dans notre pratique, en cas d'altération profonde de la fonction respiratoire en pré ou per-opératoire, la spéléotomie nous parait être une alternative intéressante à la chirurgie de résection pulmonaire majeure. Elle permet d'améliorer la survie à court et moyen terme des patients et n'exclut pas une chirurgie ultérieure redevenue possible. Cette attitude est partagée par Gebitekin et al [8] qui ont décrit la technique de cavernostomie et de myoplastie pour la chirurgie des aspergillomes complexes, suivis d'une irrigation d'amphotéricine B dans la cavité d'aspergillome: la mortalité post-opératoire était nulle dans leur série. Ono et al [13] rapportent seulement 7,1% de complications postopératoires avec la même technique au Japon. Dans notre cas, le manque d'approvisionnement continu d'antifongique de notre centre nous empêche d'inclure le paramètre traitement médical antifongique dans notre étude. D'ailleurs dans la littérature, aucune étude n'a prouvé l'efficacité du traitement antifongique par voie générale pour les patients opérés d'un aspergillome pulmonaire.

La survenue de complications respiratoires en post-opératoire était associée à une surmortalité. Dans une étude prospective, Bonde P et al [3] ont montré que la poursuite du tabagisme, la présence d'une cardiopathie ischémique, d'une bronchite chronique, ou d'un antécédent d'accident vasculaire cérébral étaient les principaux facteurs de risque de développer un encombrement bronchique après chirurgie thoracique. Dans les pays occidentaux, l'apport de la ventilation non invasive semble être prometteur, car elle permet d'améliorer la pression partielle d'oxygène de façon durable dans les suites d'une chirurgie de résection pulmonaire. Elle permet d'éviter la ventilation mécanique qui est source de pneumopathies nosocomiales, de fistules broncho-pleurales, d'un traumatisme trachéo-bronchique à l'origine d'un pneumothorax acquis sous ventilation mécanique, responsable d'une mortalité sévère de l'ordre de 8 à 28% [11]. La durée du séjour moyen en réanimation de nos patients (4 jours) est inférieure à celle rapportée de 9,5 jours par Farid et al [7]. Ceci s'explique par la plus grande fréquence de maladies concomitantes, essentiellement pulmonaires dans leur série. L'évolution à moyen et long terme des patients opérés est favorable, comme dans l'étude de Babatasi et al [2] où la survie à 10 ans était respectivement de 78% pour les formes complexes et 92% pour les formes simples. Plusieurs auteurs ont ainsi montré que la chirurgie actuelle des aspergillomes offre des résultats satisfaisants à court et long terme, avec une diminution du taux de complication, une diminution des symptômes et un taux de récidive faible [9, 12].

## Conclusion

La chirurgie reste le traitement de référence de l'aspergillome pulmonaire. La prise en charge péri-opératoire doit commencer par la recherche des facteurs de risque, ce qui permet de mieux définir les populations à haut risque, et de poser collégialement entre anesthésistes et chirurgiens l'indication d'une chirurgie d'exérèse pulmonaire.

Les facteurs de risque de complications de la chirurgie de l'aspergillose pulmonaire identifiés dans cette étude sont le score ASA, l'altération de la fonction respiratoire préopératoire, la chirurgie en urgence, le tabagisme, la ventilation mécanique postopératoire, l'hyperleucocytose, un saignement per-opératoire supérieure à 1000 ml et une durée de l'intervention supérieure à 3 heures.

En raison de la morbidité de la chirurgie de l'aspergillome pulmonaire, il est important de mettre l'accent sur la stratégie de prévention, par un dépistage précoce et un traitement optimal des cas de tuberculose pleuro-pulmonaire, afin de diminuer au maximum les séquelles parenchymateuses des patients.

## Conflits D'intérêts

Les auteurs ne déclarent aucun conflit d'intérêts.

## References

[1] Ade SS, Touré NO, Ndiaye A, Diarra O, Dia Kane Y, Diatta A, Ndiaye M, Hane AA (2011). Aspects épidémiologiques, cliniques, thérapeutiques et évolutifs de l'aspergillome pulmonaire à Dakar. Rev Mal Respir.

[2] Babatasi G, Massetti M, Chapelier A, Fadel E, Macchiarini P, Khayat A, Dartevelle P (2000). Surgical treatment of pulmonary aspergilloma: current outcome. J Thorac Cardiovasc Surg.

[3] Bonde P, McManus K, McAnespie M, McGuigan J (2002). Lung surgery: identifying the subgroup at risk for sputum retention. Eur J Cardiothorac Surg.

[4] Brik A, Salem AM, Kamal AR, Abdel-Sadek M, Essa M, El Sharawy M, Deebes A, Bary KA (2008). Surgical outcome of pulmonary aspergilloma. Eur J Cardiothorac Surg.

[5] Byun CS, Chung KY, Narm KS, Lee JG, Hong D, Lee CY (2012). Early and Long-term Outcomes of Pneumonectomy for Treating Sequelae of Pulmonary Tuberculosis. Korean J Thorac Cardiovasc Surg.

[6] Chatzimichalis A, Massard G, Kessler R, Barsotti P, Claudon B, Ojard-Chillet J, Wihlm JM (1998). Bronchopulmonary aspergilloma: a reappraisal. Ann Thorac Surg.

[7] Farid S, Mohamed S, Devbhandari M, Kneale M, Richardson M, Soon SY, Jones MT, Krysiak P, Shah R, Denning DW, Rammohan K (2013). Results of surgery for chronic pulmonary Aspergillosis, optimal antifungal therapy and proposed high risk factors for recurrence–a National Centre's experience. J Cardiothorac Surg.

[8] Gebitekin C, Sami Bayram A, Akin S (2005). Complex pulmonary aspergilloma treated with single stage cavernostomy and myoplasty. Eur J Cardiothorac Surg.

[9] Kasprzyk M, Pieczyński K, Mania K, Gabryel P, Piwkowski C, Dyszkiewicz W (2017). Surgical treatment for pulmonary aspergilloma - early and long-term results. Kardiochir Torakochirurgia Pol.

[10] Lefebvre A, Amar Y, Lorut C, Rabbat A (2014). Complications respiratoires après chirurgie pulmonaire. La Lettre du pneumologue.

[11] Lefebvre A, Lorut C, Alifano M, Dermine H, Roche N, Gauzit R, Regnard JF, Huchon G, Rabbat A (2009). Noninvasive ventilation for acute respiratory failure after lung resection: an observational study. Intensive Care Med.

[12] Lejay A, Falcoz PE, Santelmo N, Helms O, Kochetkova E, Jeung M, Kessler R, Massard G (2011). Surgery for aspergilloma: time trend towards improved results?. Interact Cardiovasc Thorac Surg.

[13] Ono N, Sato K, Yokomise H, Tamura K (2000). Surgical management of pulmonary aspergilloma. Role of single-stage cavernostomy with muscle transposition. Jpn J Thorac Cardiovasc Surg.

[14] Rakotoson JL, Razafindramaro N, Rakotomizao JR, Vololontiana HM, Andrianasolo RL, Ravahatra K, Tiaray M, Rajaoarifetra J, Rakotoharivelo H, Andrianarisoa AC (2011). Les aspergillomes pulmonaires: à propos de 37 cas à Madagascar. Pan Afr Med J.

[15] Regnard JF, Icard P, Nicolosi M, Spagiarri L, Magdeleinat P, Jauffret B, Levasseur P (2000). Aspergilloma: a series of 89 surgical cases. Ann Thorac Surg.

[16] Stéphan F (2002). Complications postopératoires de la chirurgie pulmonaire. Réanimation.

[17] Zait H, Hamrioui B (2011). Aspergillome pulmonaire: à propos de 39 cas. Journal de Mycologie Médicale.

